# The alteration of lymphocyte subsets in idiopathic granulomatous mastitis

**DOI:** 10.3906/sag-2012-192

**Published:** 2021-08-30

**Authors:** Ayça EMSEN, Hande KÖKSAL, Hülya UÇARYILMAZ, Naim KADOGLOU, Hasibe ARTAÇ

**Affiliations:** 1 Department of Pediatric Immunology and Allergy, Faculty of Medicine, Selçuk University, Konya Turkey; 2 Department of General Surgery, Ministry of Health Konya City Hospital, Hamidiye Faculty of Medicine, Sağlık Bilimleri University, Konya Turkey; 3 Department of General Surgery, London North West Healthcare NHS Trust, Northwick Park Hospital, London UK

**Keywords:** Idiopathic granulomatous mastitis, lymphocyte subsets, autoimmunity, etiopathogenesis

## Abstract

**Background and aim:**

This study analyzed peripheral blood lymphocyte subsets to determine their role in the etiopathogenesis of IGM.

**Materials and methods:**

This study includes 51 pathologically proven IGM patients (active disease: 26 and in remission: 25) and 28 healthy volunteers. The analyses of lymphocyte subsets were performed by flow cytometric immunophenotyping.

**Results:**

The percentage of T helper lymphocyte of all IGM patients were lower than control groups (p = 0.001). Absolute cytotoxic T lymphocyte count (p = 0.03), both percentage (p = 0.035) and absolute count (p = 0.002) of the natural killer cells, and both percentage (p = 0.038) and absolute count (p = 0.008) of natural killer T cells, were higher than the control group. The T helper lymphocyte percentage of the patients with active disease was lower than the control group (p = 0.0003). The absolute cytotoxic T lymphocyte (p = 0.029) and natural killer T cells (p = 0.012) of the patients with active disease were higher than the control group.

**Conclusion:**

Idiopathic granulomatous mastitis is defined as a localized form of granulomatous disorders. However, the observed changes in T cells, NK, and NKT cells suggest that there is systemic immune dysregulation in patients with IGM.

## 1. Introduction

Idiopathic granulomatous mastitis (IGM), a benign inflammatory disease, is rarely seen worldwide. However, it is more frequent in some Middle Eastern countries such as Turkey, Egypt, and Iran and Asian countries such as China, India, and Pakistan. Currently, no etiological factors accused, including autoimmune disorders, rheumatologic diseases, infectious causes, and hormonal imbalances, could be proven. Granulomatous inflammation can be seen in various diseases of the breast, but the most common form is idiopathic in especially these countries [1–4]**.**

Remission and relapse of IGM in some patients with rheumatologic findings, such as arthritis and/or erythema nodule, suggest autoimmunity and immune dysregulation [5–11]. Erhan et al. [12] found T lymphocyte domination in the tissue of IGM patients. Similarly, in the study by Chen et al. [13], the predominance of CD3+ cells were detected higher than CD20+ cells in breast granulomatous tissue immunohistochemically. The high incidence of some human leukocyte antigens in patients with IGM also shed light on the etiopathogenesis [14].

Lymphocytes and their products constitute the most important part of the immune system. The main subgroups of lymphocytes are T-cells, B-cells, and natural killer (NK) cells. In order to establish and maintain a normal immune response, there must be a balance between the lymphocyte subgroups with regulatory and effector functions. There are almost no studies on autoimmunity considering it as an etiologic factor in IGM patients who may have abnormalities in humoral and cellular immune systems [15]. 

In this study, the changes of peripheral blood lymphocyte subsets in IGM patients and its possible role in pathogenesis were aimed to be investigated.

## 2. Methods

### 2.1. Patients

Between March 2019 and May 2020, 51 patients with pathologically proven IGM and 28 healthy volunteers, as the control group, were enrolled. The IGM patients were divided into active IGM, and in remission IGM according to the activity of their disease. Active IGM (n: 26) was defined as patients with clinically and radiologically defined lesions with pathological diagnosis, whilst patients in remission (n: 25) had no clinical or radiological symptoms and findings except for their former pathological diagnosis. The criteria defined by Kessler and Wolloch [16] and detailed by Cohen [17] were used in the pathological diagnosis.

Demographic data including age, marital status, whether the birth, the number of children, breastfeeding history, and use of oral contraception, symptoms, and signs were noted.

**Flow cytometric analysis**

The blood samples were collected during the first admission, before any treatment was administered in active IGM patients, whereas the samples were collected at least six months after the termination of treatment in patient with remission. In order to evaluate the lymphocyte subgroups, Using EDTA tubes, 2 mL blood samples were collected from patients and delivered to the pediatric immunology laboratory. Peripheral blood samples were collected in K2-EDTA anticoagulant. One hundred µL of anticoagulated whole blood was aliquoted into 5 mL polystyrene falcon flow cytometer tubes (Becton Dickinson, New Jersey, USA) and stained with antihuman CD3-PE-Cy5, CD4-PE, CD8-FITC, CD19-APC, CD16+CD56-PE, CD45-APC monoclonal antibodies (Biolegend, San Diego, USA) at the manufacturer’s recommended concentration. The tubes were incubated in the dark for 20 min at room temperature. Red blood cells were lysed by adding 2 mL 1X red blood cells lysis buffer (Biolegend, San Diego, USA) and incubating in the dark at room temperature for 10 min. Then the tubes were centrifuged for 5 min at 1500 rpm. The cell pellet was washed once and re-suspended in 500 μL cell staining buffer (Biolegend, San Diego, USA). Lymphocyte subset analysis was performed using a FACS Aria III flow cytometer (Becton Dickinson, USA) and FACS Diva v: 6.1.3 Software (Becton Dickinson, New Jersey, USA) within 24 h. Lymphocyte subsets were identified from lymphocyte population as Total T cells (CD3^+^), T helper cells (CD3^+^CD4^+^), cytotoxic T cells (CD3^+^CD8^+^), B cells (CD19^+^), natural killer cells (NK-CD3^-^CD16^+^56^+^), and natural killer T cells (NKT-CD3^+^CD16^+^56^+^).

**Statistical analysis**

SPSS-15 software was used for all statistical analyses. For numerical data, mean ± standard deviation (SD) or median (min-max) were used according to the distribution of data. The Kruskall–Wallis test was used to compare the ages of the groups. Student t test was used to compare the means of two independent groups. In comparing the means of more than two independent groups, one-way ANOVA was used. At one-way ANOVA, when a significant difference was found, Tukey post-hoc analysis was used for comparing groups. In the comparison of categorical data, Fischer Exact test was used as the data did not meet the assumptions required for χ^2^ test.

An alpha value (p) less than 0.05 was considered statistically significant.

## 3. Results

Fifty-one IGM patients, and 28 healthy volunteers as the control group were enrolled. The median age of patient and control groups were 37 (26–64 years), and 34 years (20–52 years) respectively. There was no statistically significant difference in age between the patients and healthy volunteers (p = 0.31). Demographic and clinical features of all the patients enrolled and the treatment approaches are given in Table 1.

**Table 1:** The patients’ clinical features.

	With active disease(n = 26)	In remission†(n = 25)	p-value
Parity status*			
Nulliparous	0 (0)	1 (4)	0.999
Parous	25 (100)	24 (96)	
Breastfeeding history*	25 (100)	24 (96)	0.999
Oral contraceptive use history	3 (12)	7 (28)	0.15
Smoking*	2 (8)	3 (12)	0.999
Menopausal status*			
Premenopausal	23 (92)	25 (100)	0.49
Postmenopausal	2 (8)	0	
Symptom and signs			
Mass*	25 (100)	24 (96)	0.66
Pain*	23 (92)	21 (84)	0.999
Erythema	16 (64)	16 (64)	0.999
Abscess	12 (48)	15 (60)	0.39
Axillary lymphadenopathy	13 (52)	8 (32)	0.25
Peau d’orange	2 (8)	2 (8)	0.999
Nipple retraction*	0	4 (16)	0.11
Ulcer*	3 (12)	2 (8)	0.999
Fistula*	0	3 (12)	0.11
Erythema nodosum*	1 (4)	3 (12)	0.6
Treatment approaches	**		
Wait and watch		3 (12)	
Only antibiotics		4 (16)	
Drainage + antibiotics		9 (36)	
Intralesional steroid		5 (20)	
Systemic steroid		3 (12)	
Topical and intralesional steroid		1 (4)	

† For idiopathic granulomatous mastitis patients in remission clinical features at the time of first diagnosis are given. At the time of study these patients had no symptoms or findings.

**Comparison of control group and all patients’ lymphocyte subsets**

The absolute counts of leukocytes, neutrophils and monocytes in IGM patients were higher than in the control group. While lymphocyte percentage and T helper lymphocyte percentage were statistically lower in the patient group compared to the control, lymphocyte absolute count, T cytotoxic lymphocyte absolute count, natural killer cell percentage and absolute count and natural killer T cell percentage and absolute count were statistically higher in the patient group compared to the control group. T lymphocyte count and percentage, T helper lymphocyte absolute count, T cytotoxic lymphocyte percentage, total B lymphocyte percentage and absolute count, and double negative T cell percentage and absolute count were similar between the two groups. In addition, the CD4/CD8 ratio was found to be statistically lower in the patient group compared to the control group. The absolute count and percentage of leukocytes, neutrophils, monocytes, lymphocytes, the lymphocyte subset values, and CD4/CD8 ratio were given in Table 2.

**Table 2:** The patients and control groups’ lymphocyte subsets.

	MAIN GROUPS		
	All IGM patients(n=51)	Control groups(n=28)	p
Leukocyte counts	8810.4 ± 2694.8	6824.2 ±1583.9	.001
Neutrophil			
%	61.46 ± 7.64	57.61± 5.08	.019
Absolute counts	5530.1 ± 2096.3	3946.3 ± 1020.3	< .0001
Monocyte			
Absolute counts	530.7 ± 258.9	408.2 ± 116.4	.005
%	5.91 ± 1.52	6.07 ± 1.51	.64
Lymphocyte			
%	27.85 ± 7.04	31.22 ± 5.22	.015
Absolute counts	2362.72 ± 702.99	2119.61 ± 531.82	.11
Lymphocyte subsets			
Total T lymphocyte			
%	74.22 ± 7.97	77.53 ± 5.56	.055
Absolute counts	1742.44 ± 513.46	1641.78 ± 420.49	.38
T helper lymphocyte			
%	42.87 ± 7.67	48.83 ± 5.56	.001
Absolute counts	1008.05 ± 343.49	1033.74 ± 275.30	.73
T cytotoxic lymphocyte			
%	25.08 ± 6.13	23.05 ± 5.23	.14
Absolute counts	585.51 ± 196.45	488.78 ± 175.81	.03
Natural killer cells			
%	13.9 ± 6.95	10.74 ± 4.7	.035
Absolute counts	332.88 ± 190.03	225.76 ± 104.5	.002
Total B lymphocyte			
%	9.64 ± 3.86	9.16 ± 3.75	.59
Absolute	228.85 ± 127.1	200.63 ± 110.96	.32
Natural killer T cell			
%	5.92 ± 3.41	4.53 ± 2.38	.038
Absolute counts	144.33 ± 111.11	94.17 ± 49.95	.008
Double negative T cells			
%	6.38 ± 3.48	5.64 ± 3.14	.35
Absolute counts	151.58 ± 91.59	119.34 ± 71.22	.11
CD4/CD8 ratio	1.85 ± 0.77	2.26 ± 0.74	.023

**The comparisons of control group and the subgroups of patients**

The absolute count and percentages of leukocytes, neutrophils, monocytes, and lymphocytes, the lymphocyte subset values of the control group and the subgroups of IGM patients were given in Table 3. The leukocyte counts and absolute counts of neutrophils, T cytotoxic lymphocytes, NK and NKT cells were higher in patients with active IGM disease than the control group. The percentages of neutrophils, T cytotoxic lymphocytes, NK and NKT cells were also statistically higher in patients with active IGM than the control group (Table 3). The percentages of lymphocytes and T helper lymphocytes were statistically lower in patients with active disease than the control group. CD4/CD8 ratio was lower in patients with active disease when compared with the control group. 

**Table 3:** The subgroups and control groups’ hematological parameters and lymphocyte subsets.

	SUBGROUPS			p
	With active disease(n=26)	In remission(n=25)	Control groups(n=28)	
Leukocyte counts	9545.0 ± 2214.8	8077.0 ± 2995.0	6824.2 ±1583.9	< .0001a
Neutrophil				
%	63.58 ± 7.68	5979.0 ± 6.69	57.61 ± 5.08	.005b
Absolute counts	6093.2 ± 1744.4	4920.25 ± 2304.4	3946.3 ± 1020.3	<.0001c
Monocyte				
%	5.59 ± 1.67	6.21 ± 1.33	6.07 ± 1.51	.277
Absolute counts	543.7 ± 229.5	516.6 ± 291.9	408.2 ± 116.4	.06
Lymphocyte				
%	26.5 ± 6.72	29.26 ± 7.22	31.22 ± 5.22	.02d
Absolute counts	2503.5 ± 800.7	2210.21 ± 556.16	2119.61 ± 531.82	.08
Lymphocyte subsets				
Total T lymphocyte				
%	74.03 ± 6.84	74.42 ± 9.15	77.53 ± 5.56	.15
Absolute counts	1833.62 ± 537.6	1643.67 ± 477.45	1641.78 ± 420.49	.25
T helper lymphocyte				
%	41.9 ± 5.85	43.89 ± 9.21	48.83 ± 5.56	.002e
Absolute counts	1047.13 ± 346.91	965.72 ± 342.0	1033.74 ± 275.30	.64
T cytotoxic lymphocyte				
%	25.57 ± 6.01	24.58 ± 6.34	23.05 ± 5.23	.28
Absolute counts	624.4 ± 210.78	543.37 ± 174.26	488.78 ± 175.81	.03f
Natural killer cells				
%	13.5 ± 6.54	14.27 ± 7.46	10.74 ± 4.7	.1
Absolute counts	347.72 ± 208.59	316.82 ± 170.66	225.76 ± 104.5	.02g
Total B lymphocyte				
%	9.47 ± 4.43	9.82 ± 3.25	9.16 ± 3.75	.82
Absolute counts	240.44 ± 154.84	216.31 ± 89.66	200.63 ± 110.96	.48
Natural killer T cell				
%	6.65 ± 3.36	5.16 ± 3.36	4.53 ± 2.38	.039 h
Absolute counts	168.36 ± 104.73	118.3 ± 114.1	94.17 ± 49.95	.015i
Double negative T cells				
%	6.81 ± 3.7	5.92 ± 3.25	5.64 ± 3.14	.42
Absolute counts	167.85 ± 96.54	133.97 ± 84.38	119.34 ± 71.22	.1
CD4/CD8 ratio	1.74 ± 0.55	1.96 ± 0.94	2.26 ± 0.74	.045j

a With active disease & control group, p < .00001.b With active disease & control group, p = 0.004.c With active disease & control group, p < 0.0001.d With active disease & control group, p = 0.033.e With active disease & control group, p = 0.0003; in remission & control groups, p = 0.054.f With active disease & control group, p = 0.029. g With active disease & control group, p = 0.023.h With active disease & control group, p = 0.034.i With active disease & control groups, p = 0.012.j With active disease & control groups, p = 0.036.

## 4. Discussion

Although some infectious causes and autoimmune processes, diabetes mellitus, and sarcoidosis can cause granulomatous reactions, the most common form of granulomatous inflammation in the breast is idiopathic [1]. In the present, the etiology of IGM is not clear. However, different factors including the deficiency of alpha-1 antitrypsin, oral contraceptives, smoking, hyperprolactinemia, ethnicity, autoimmunity, gestation, and birth and breast-feeding have been thought to be involved in the etiopathogenesis of IGM [1,2]. Recently, autoimmunity and immune dysregulation have been emphasized, but studies on this subject are limited.

Some patients with IGM respond well to immunosuppressive drugs such as steroids and methotrexates; the presence of extramammary findings likes erythema nodosum or arthritis in some patients and some recent studies, although limited, suggest that more emphasis should be given to autoimmunity and immune dysregulation in IGM [3–13]. The aim of our study was to investigate the number of T and B lymphocytes and changes in NK and NKT cells in IGM patients. 

In the study by Altıntoprak et al. [3], they investigated the autoantibodies including ANA and ENA levels in IGM. However, the authors could not demonstrate any relationship between autoimmunity and IGM. In another important study, the authors serologically examined rheumatoid factor, ANA and anti-dsDNA in eight patients with IGM. Rheumatoid factor positivity was detected in 6 patients and ANA and anti-ds DNA positivity were detected in 2 patients. In these two patients, rheumatoid factor, ANA, and anti-ds DNA were all positive [18]. Unfortunately, in our article on autoantibodies in IGM, the newly online ahead of print, our findings did not support the clinical utility of autoantibodies including RF, ANA, anti-ds-DNA, pANCA and anti-CCP in IGM neither in diagnosis nor in follow up [19]. 

In a very important study by Erhan et al. [12], the authors investigated T and B lymphocyte markers in biopsy specimens in patients with IGM. In this study, T lymphocyte predominance was observed in biopsy specimens. Similarly, in the study by Chen et al. [13], the predominance of CD3^+^ cells were detected higher than CD20^+^ cells in breast granulomatous tissue immunohistochemically.

In our previous study, cytokine changes in patients with IGM were examined. The levels of interleukins -8, -10, and -17 in patients with IGM were found to be higher than the controls. We concluded that interleukins-8 and -17, proinflammatory cytokines, could have a role in the pathogenesis of IGM, but the low interleukin-10 levels suggested the reduction in the release of proinflammatory cytokines as well as suppressing their function. Therefore, it contributes to controlling the extent of disease [20].

Granulomatous inflammation, a form of delayed type hypersensitivity reaction, is a protective response to chronic infections with persistent pathogens such as mycobacterial infections. Granuloma formation may appear as a primary lesion in cases where the cause is unknown, as in sarcoidosis or IGM. In addition, the important role of T helper cells in granuloma formation is well known [21]. Similar findings were found in the studies by Erhan et al. [12] and Chen et al. [13]. In our study, T helper cell ratio of all the IGM patients were lower than the control group. In subgroups analyses, T helper cell ratio of the patients with active disease was lower. Recruitment of T helper cells in granulomatous breast tissue may be a reason of low T cells in peripheral blood of the patients. 

The role of T cytotoxic cells in some granuloma formation including listeriosis is known and T cytotoxic cells’ accumulation are shown in effector phase of granuloma formation [22]. In this experimental study, listeriosis leading to rapid activation, proliferation and apoptosis of CD4 + and CD8 + T cells were revealed. In another study by Mannering and Cheers, chronic *Mycobacterium avium* infection was found to be associated with an increase in T-cell apoptosis and elevated, but sustained levels of in vivo proliferation [23]. In our study, absolute cytotoxic T cell count was found to be high in active IGM patients. This increase of T cytotoxic cells and the change of CD4/CD8 ratio may be related to a decrease in T helper cells. The limitation of our study was the lack of longitudinal follow-up of lymphocyte subsets in active patients.

Natural killer T cells play an important role in suppressing granuloma formation in the liver of mice by modulating the production of IFN-ɣ and IL-10. In this experimental study, they revealed increased granuloma formation in mice without NKT cells, which may be explained by an increased interferon gamma production. As a result, NKT cells were found to suppress granuloma formation [24]. We found that the NK cells and NKT cells were higher in IGM patients than the control group. The present results suggest that NKT cells may play a role in regulating and/or suppressing granuloma formation. 

In conclusion, understanding the regulatory capacity of T cell subsets and NK cells in granuloma formation in IGM may allow understanding of the etiophatogenesis and lead to the development of new therapeutic agents for this disease which is full of secrets. 

## Funding

This study was supported by Selçuk University the Scientific Research Projects of Selçuk University (Project No: 18401172). 

## Informed consent

Local ethics committee of Selçuk University approval (No: 2018/171 on 02 May, 2018) and the written informed consent from patients were obtained.
